# A Literature Review on Early Kangaroo Mother Care: Historical Roots, Neonatal Intensive Care Unit (NICU) Integration, and Challenges

**DOI:** 10.7759/cureus.85140

**Published:** 2025-05-31

**Authors:** Purnima Sahoo, Santosh K Panda, Niyati Das

**Affiliations:** 1 Pediatrics, Kalinga Institute of Nursing Sciences, Kalinga Institute of Industrial Technology (KIIT) Deemed To Be University, Bhubaneswar, IND; 2 Neonatology, Kalinga Institute of Medical Sciences, Kalinga Institute of Industrial Technology (KIIT) Deemed To Be University, Bhubaneswar, IND

**Keywords:** breastfeeding, early kangaroo mother care, infant mortality, low-birth-weight babies, neonatal intensive care unit, skin-to-skin contact

## Abstract

Kangaroo mother care (KMC), which includes skin-to-skin contact (SSC) and exclusive breastfeeding, has become a widely recognized and cost-effective method for improving the health and survival of preterm and low-birth-weight (LBW) infants. This review explores the evolution of implementing early KMC, its integration into neonatal intensive care units (NICUs), and the barriers hindering its widespread adoption. The benefits of early KMC include better temperature regulation, improved breastfeeding, stronger parent-infant bonding, and reduced mortality. However, many hospitals and communities still face challenges, including a lack of awareness, limited training among healthcare staff, cultural resistance, and inadequate infrastructure. To overcome these obstacles, this review emphasizes the importance of supportive policies, parental education, and ongoing training for healthcare providers. It also discusses the need for practical models and implementation strategies to help expand early KMC programs at both the local and national levels. Drawing on global experiences and new data from countries that have successfully scaled up early KMC, this review presents a vision in which families play a central role in caring for their newborns from birth. Making early KMC available to all who need it, both in hospitals and in the community, is essential for improving newborn survival and achieving global health targets, including the Sustainable Development Goals. This review calls for stronger collaboration among researchers, policymakers, and healthcare workers to establish early KMC as a standard practice in newborn care worldwide.

## Introduction and background

Globally, around 18% of preterm neonates require hospitalization in a neonatal intensive care unit (NICU), with this rate rising to about 33% in underdeveloped nations [1,2]. Prematurity is a major contributor to neonatal mortality and morbidity, and around 30% of neonatal mortality in underdeveloped nations is secondary to preterm birth [3]. Infants born preterm (<37 weeks of gestation) and with low birth weight (LBW) (<2,000 g) are at increased risk for various neonatal morbidities, including respiratory, gastrointestinal, immunologic, central nervous system, hearing, and vision problems. These risks are significantly higher compared to those faced by full-term, normal-weight infants [4].

Kangaroo mother care (KMC), defined as skin-to-skin contact (SSC) between a mother and her newborn, has been shown to promote exclusive breastfeeding, facilitate early hospital release, and reduce neonatal mortality among preterm and LBW infants, regardless of economic status or geographical location [5-7]. Although initiating early KMC during the first week of life can be challenging due to neonatal illness, studies have demonstrated its feasibility even for preterm infants receiving respiratory support through ventilators, continuous positive airway pressure (CPAP), or nasal cannula [8,9]. Providing early KMC to ventilated newborns typically requires a 1:1 nurse-to-patient ratio, which is often difficult to maintain in resource-limited settings. Several studies have shown that early initiation of KMC supports lactation by helping the baby learn to root and latch effectively [10,11], resulting in significantly higher rates of exclusive breastfeeding compared to late KMC. Furthermore, early KMC has been associated with a marked reduction in neonatal mortality among ill preterm infants, from 38% to 22.5% [12,13]. Additionally, the WHO recommends initiating KMC after clinical stabilization. However, stabilization may take hours or even days, depending on the infant's gestational age, birth weight, and overall condition at birth. Facility-based studies have reported that the median age at initiation of KMC ranges from 3 to 24 days [14]. Delayed initiation (beyond the first three days of life) may limit its impact on early neonatal mortality, as approximately 62% of neonatal deaths occur within the first three days of life [15]. Studies confirm that early KMC is safe and offers additional benefits, including a shorter time to full enteral feeds, improved nutritional outcomes in moderately ill preterm neonates, and increased survival among mildly to moderately unstable neonates [16,17]. 

## Review

Objectives, scope, and methodology of the literature review

**Figure 1:**
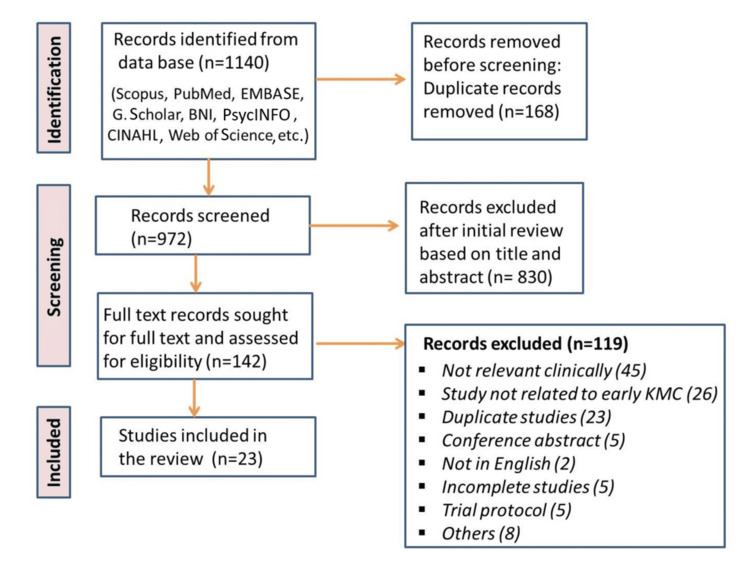
Schematic representation of the database search strategy and article selection process

This literature review evaluates the early initiation of KMC (defined as within 24 hours of birth) in NICUs from its inception in the early 2000s through 2024, and examines its impact on clinical outcomes, including breastfeeding, growth, mortality, and morbidity. All trials involving early initiation of KMC in LBW or preterm infants were included. An online search was conducted across multiple databases, including Scopus, PubMed, Google Scholar, EMBASE, BNI, PsycINFO, CINAHL, and Web of Science, to identify relevant scholarly articles. Key search terms included "kangaroo mother care", “early KMC benefits”, “skin-to-skin contact", “low birth weight baby", "neonate", and "infant child development". These terms were used individually and in combination, applying Boolean operators (AND, OR, NOT) to refine results. A total of 142 articles were retrieved for full-text review and assessed for eligibility. Of these, 23 articles were included in the final review after excluding 119 records deemed irrelevant (Figure 1).

Studies were excluded if they did not meet the predefined inclusion criteria, such as lacking primary data, being non-English, or not focusing on the target population or intervention. Duplicate studies were identified through title and abstract screening and were removed accordingly. Quantitative studies and guidelines were included in the review based on their relevance to the research objectives. Quantitative studies contributed empirical data essential for evidence synthesis, while guidelines were included for their expert consensus and practical recommendations, which complement empirical findings and inform best practices.

A list of articles related to early KMC was compiled and is presented in Table 1. Case reports were not evaluated. The primary objective of this review was to assess the impact of early KMC on newborn and infant mortality, as well as severe morbidities, among LBW and preterm infants. Specifically, the review focuses on (a) the implementation of early KMC in NICUs and (b) the challenges associated with its adoption. The findings of this review are intended to support policymakers and other stakeholders by providing critical evidence that may inform the development of clinical practice guidelines and strategies for scaling up early KMC.

**Table 1:** Summary of articles related to early KMC included in this study

References	Aim/scope	Study type/design	Country of study
Tharashree et al. [7]	Early versus late KMC for stable low-birth-weight infants	Systematic review	Japan
Worku and Kassie [13]	A randomized controlled trial on the effectiveness of early KMC for low-birth-weight infants	Quantitative study	Addis Ababa, Ethiopia
Ayele and Antenen [16]	The proportion of mothers who continued to practice kangaroo mother care at home and identify potential factors influencing this practice following hospital discharge	Quantitative study	Ethiopia
Madiba and Sengane [17]	Tube feeding practices and transition to breastfeeding experiences of mothers of preterm infants at a kangaroo mother care unit	Quantitative study	South Africa
Brotherton et al. [18]	The beneficial effect of early and prolonged kangaroo mother care on long-term neurodevelopmental outcomes in low-birth-weight neonates: a cohort study	A cohort study	India
Chwo et al. [19]	Early kangaroo care for preterm infants: effects on temperature, weight, behavior, and acuity	Quantitative study	Taiwan
Mörelius et al. [20]	To describe the time of first skin-to-skin contact in extremely preterm infants from a national perspective	Quantitative study	Sweden
Nimbalkar et al. [21]	Effect of early skin-to-skin contact following normal delivery on the incidence of hypothermia in neonates weighing more than 1800 g	Randomized controlled trial	India
Moore et al. [22]	Early skin‐to‐skin contact for mothers and their healthy newborn infants	Qualitative study	USA
Purushotham et al. [23]	Feasibility and efficacy of early KMC in very low birth weight babies receiving noninvasive respiratory care in NICU	Qualitative study	India
Jayaraman et al. [24]	Effect of early KMC versus late KMC on human milk feeding in low-birth-weight neonates	Quantitative study	India
Ayele et al. [25]	Kangaroo mother care (KMC) comprises early and continuous skin-to-skin contact between mother and baby as well as exclusive breastfeeding	Quantitative study	Ethiopia
Pandya et al. [26]	The beneficial effect of early and prolonged kangaroo mother care on long-term neuro-developmental outcomes in low birth weight neonates	A cohort study	India
WHO Immediate KMC Study Group [27]	Immediate "kangaroo mother care" and survival of infants with low birth weight	Quantitative study	Global
Mörelius et al. [28]	A randomized trial of continuous skin-to-skin contact after preterm birth and the effects on salivary cortisol, parental stress, depression, and breastfeeding	A randomized controlled trial	Sweden
Nagai et al. [29]	Early versus late continuous kangaroo mother care (KMC) for stable low-birth-weight infants: a randomized controlled trial	A randomized controlled trial	Japan
Pandya et al. [30]	Early kangaroo mother care in preterms weighing ≤1250 grams: before and after a training program in neonatal nurses	Quantitative study	India
Ludington-Hoe et al. [31]	Birth-related fatigue in 34-36 week preterm neonates; rapid recovery with very early kangaroo (skin to skin) care	Quantitative study	USA
Whitelaw et al. [32]	Skin-to-skin contact for very low birth weight infants and their mothers	Quantitative study	UK
Forde et al. [33]	Oxidative stress biomarkers decreased in preterm neonates treated with kangaroo mother care	Quantitative study	USA
The American Academy of Pediatrics [34]	Immediate kangaroo mother care (iKMC) by the American Academy of Pediatrics	Guidelines	Global
Hull and Gongora [35]	Implementing early kangaroo mother care: a quality improvement initiative	Quality improvement initiative studies	USA
Talikoti et al. [36]	Skin-to-skin contact for infants: guidelines for professionals by Children’s Hospital of Philadelphia	Guidelines for professionals	USA

Historical background

Origin and Evolution of Early Kangaroo Mother Care

KMC was first introduced in 1978 by Dr. Edgar Rey in Bogotá, Colombia, as a response to overcrowding and a shortage of incubators for preterm infants [37]. Initially, KMC consisted of SSC, exclusive breastfeeding, and early discharge, aiming to improve outcomes for LBW infants. Over time, research on KMC expanded globally. Early randomized controlled trials conducted in the 1990s and 2000s demonstrated significant reductions in mortality, infections, and hospital stays [38]. Systematic exploration of very early initiation of KMC (within the first 24 hours of birth) emerged more prominently in the early 2000s and gained momentum after WHO’s 2015 recommendations promoting integration of KMC into neonatal care pathways [1,39]. Recent trials, such as the WHO’s iKMC study in 2021, have provided robust evidence that initiating KMC immediately after birth, even before full clinical stabilization, can further reduce neonatal mortality among unstable preterm newborns [40]. These findings represent a significant shift from traditional practices and support the adoption of early KMC as a critical intervention for improving survival outcomes among preterm and LBW infants worldwide [27].

Every year, approximately 15 million infants are born preterm and often require hospitalization in a NICU for specialized care and monitoring [41]. For families, hospitalization of a premature newborn is often a stressful and unexpected experience, as neonates are separated from their mothers at birth and placed in a highly medicalized and unfamiliar environment [42]. This disruption highlights critical healthcare challenges, resulting in low breastfeeding rates of 22.5% for preterm newborns, well below the WHO's recommendation of exclusive breastfeeding for the first six months of life. Low breastfeeding rates negatively impact both maternal and neonatal health by limiting opportunities for SSC and increasing the risk of infant morbidity and mortality [43,44]. 

Initially, KMC was offered to LBW infants weighing less than 2,000 g who were able to breathe and feed independently [39]. KMC is not contraindicated by intravenous infusions, monitor leads, pulse oximetry, supplemental oxygen, nasal ventilation, or cardiopulmonary monitoring. Infants receiving KMC exhibit stable oxygen requirements and a lower incidence of bradycardia and apnea [45,46]. Recent studies have also shown that early and prolonged KMC is associated with improved neurodevelopmental outcomes, particularly in cognition and adaptive behavior domains [47].

The benefits of KMC were first systematically explored in 1994 within resource-limited settings, emphasizing the kangaroo position and feeding as practical alternatives to advanced neonatal technologies. Subsequently, research on KMC grew, rapidly expanding in both developed and developing nations during the mid-1990s, with numerous studies reporting the effects of KMC on mortality, morbidity, and quality of survival among LBW infants [48]. Data from multiple studies consistently indicate that KMC significantly reduces the risk of neonatal death, hospital-acquired infections, and hypothermia. It is also associated with increased rates of exclusive breastfeeding, improved weight gain, and enhanced linear growth [38].

Introduction and importance of early KMC

Modern, evidence-based research has demonstrated that early KMC provides numerous benefits for preterm and LBW infants, including (i) reduced infant mortality and decreased risk of hospital-acquired infections, including sepsis (a serious systemic infection common in small and preterm neonates); (ii) improved infant weight gain; (iii) increased breastfeeding rates; (iv) enhanced neurodevelopmental and neuromotor outcomes; and (v) strengthened mother-infant bonding, among others. These outcomes have been documented over several decades of research, much of which has been conducted by investigators in emerging economies [38]. Early KMC is crucial because it should begin as soon as possible after birth, even before the baby is completely stabilized, and should be continued consistently both before and after stabilization [45,46].

Implementation of early KMC in the NICU

Successful implementation of early KMC within NICUs requires a paradigm shift from traditional incubator-based care to a family-centered, SSC model. Emerging evidence, including data from the iKMC trial and other large-scale studies, indicates that early initiation of continuous KMC can reduce neonatal mortality by up to 25%, even among clinically unstable preterm infants [5,9]. Key components for effective implementation include timely identification of eligible infants, comprehensive training for healthcare providers, parental education, and modification of NICU protocols to prioritize SSC over mechanical interventions when clinically appropriate [49]. Furthermore, recent WHO implementation guidelines recommend integrating early KMC into standard newborn care policies. These guidelines emphasize the importance of collaborative efforts between families and healthcare teams to address challenges related to staffing limitations, inadequate infrastructure, and cultural resistance [1,39]. Thus, a structured, evidence-based approach is essential for scaling up early KMC practices globally and optimizing neonatal outcomes.

To support the successful implementation of KMC, hospitals should ensure 24-hour parental access to the neonatal unit. Dedicated KMC spaces, next to or at the neonatal unit, should be equipped with comfortable seating options such as reclining chairs in the postpartum and nursery wards, as well as beds with adjustable backrests for mothers. With the aid of pillows, mothers can maintain a semi-reclining position while providing KMC, either in bed or in a standard chair. Comprehensive KMC training for nursing staff is essential and should cover key areas including nutrition for LBW infants, breast milk expression and storage, alternative feeding methods, and daily monitoring of infant growth [50]. Mothers, families, and others should have access to educational resources on KMC in the local language, including information sheets, posters, and video films. Implementation of KMC should follow a structured process comprising three key phases: pre-implementation, implementation, and follow-up, to ensure sustained success and benefit for newborns [51].

Early KMC and breastfeeding in LBW babies

Information about KMC and the critical importance of appropriate care for LBW and preterm newborns can be effectively integrated into various community programs and outreach initiatives to raise awareness and promote best practices among families and caregivers. Early KMC is anticipated to improve nursing practices and health outcomes for LBW newborns. In Madagascar, studies assessing the effects of early continuous KMC on exclusive breastfeeding at six months showed that while early KMC initiation provided some benefit, neither early nor late initiation had a substantial impact on short- or long-term breastfeeding outcomes when compared [51,52]. Therefore, the present study highlights the comparative effects of early versus late KMC initiation on breastfeeding rates among LBW infants, particularly focusing on sick but clinically stable neonates still receiving oxygen, CPAP, or intravenous fluids. There has not been any research on whether breastfeeding rates would increase if ill babies received KMC sooner [1]. Jayaraman et al. revealed that early KMC significantly improved exclusive human milk feeding rates in very-LBW neonates compared to late KMC initiation [24]. This suggests that early KMC can be safely initiated in unwell but stable very-LBW newborns, even while receiving respiratory support. In this study, infants admitted to level 2 or 3 neonatal care units within the first four days of life were randomly assigned to receive either late KMC (after complete stabilization, defined as cessation of intravenous fluids and respiratory support) or intermittent early KMC, which was started shortly after randomization. While no significant differences were observed in mortality or growth parameters between the groups, early KMC had a clear positive impact on human milk feeding practices during hospitalization and after discharge [52]. Initiation of KMC was feasible even within 24 hours of birth and at a median (interquartile range) of 3.2 (1-8) days, including among mothers who delivered via cesarean section [53]. According to Rojas et al. [54], KMC in very-LBW infants (<1,501 g, <32 weeks of gestation) was initiated at a mean of 19 days while they were still receiving ventilator support, CPAP, or oxygen via nasal cannula [9]. Notably, many studies excluded mechanically ventilated neonates due to safety concerns and the inability to maintain a 1:1 nurse-to-patient ratio in resource-limited settings [1]. Nevertheless, data from the study indicated that a significantly higher proportion of neonates in the early KMC group were exclusively breastfed and received human milk during hospitalization as well as post-discharge [24]. According to Widström et al., early initiation of KMC supports lactation by teaching the baby how to root and latch onto the breast [10]. When they evaluated the proportion of exclusive breastfeeding at six months postnatal age between early and late KMC initiation, they observed a significant difference [7]. Hospitals that implemented rooming-in and early kangaroo care in the postpartum phase observed increased breastfeeding rates among postpartum mothers [12]. Early initiation of KMC in ill infants significantly reduced mortality to 22.5%, compared to 38% with standard treatment [13]. Extended duration of KMC was also associated with improved breastfeeding continuation at three and four months, especially in extremely preterm infants (<32 weeks of gestation) [55]. 

Efforts to improve awareness about KMC and the importance of appropriate care for LBW and preterm newborns are increasingly being integrated into existing community programs and events. In the past, neglect and a lack of KMC practices were the most frequent causes of infection and hypothermia in infants. However, educating communities on effective, evidence-based practices like early KMC, exclusive breastfeeding, infection prevention, and thermal protection may improve survival and developmental outcomes [56]. The majority of mothers' positive sentiments toward fostering stronger interactions and bonds with their newborns can be explained by KMC. Nonetheless, more than half of the moms reported feeling uncomfortable and worried about heat and perspiration as "uncomfortable factors." Hence, KMC should be incorporated, with proper monitoring, assessment, and evaluation, into fundamental policies pertaining to newborn, maternal, and child health at the national level [57].

Further investigation is required to assess the impact of KMC on the development and physiologic stability of infants. The KMC babies were heavier, had bigger heads, and showed superior neurobehavioral development. Heart rate (HR), respiratory rate (RR), oxygen saturation rate (O_2_ sat), and body temperature can all remain consistent in infants who are clinically stable, preterm, or severely preterm. During KMC in the early postpartum period, infants maintain a steady body temperature [58]. KMC prolongs sleep, which includes peaceful rest intervals. Most clinicians learn KMC's practices through direct observation or experience. Many lacked proper training and confidence when it came to treating LBW with KMC, which also had to do with the safety of the infant.

KMC is beneficial because it helps LBW newborns stabilize their HR by fortifying the mother-child relationship. This finding emerged during specific trials where mothers were asked to practice KMC while they were hospitalized, and LBW infants were monitored. Furthermore, clinical experts believe that placing LBW infants in KMC facilitates early access to breast milk, enabling both mother and infant to promote quicker weight gain [59]. KMC promotes bonding, increases a mother's ability to produce breast milk, and moves the milk supply closer to and more easily accessible for the baby [60].

Benefits of early KMC in the NICU

KMC mitigates hypothermia by transferring warmth from the mother to the infant and enhances stability, especially in infants born extremely prematurely. In addition, there is less volatility in cardiac and respiratory rates, such as oxygen saturation, fewer bradycardia and apnea events, and reduced pain response among premature newborns during uncomfortable operations [61,62]. It also lowers the secretion of cortisol; reduces stress, which promotes the babies' sleeping habits and brain development; and improves psychomotor and neurobehavioral development [63,64]. KMC sessions raise psychological adaptation, recovery during preterm delivery, psychological well-being and foster communication between parents and babies [65]. It facilitates access to the breast and enhances human milk production, increases breastfeeding rates, improves the proportion of exclusive breastfeeding at NICU discharge, and supports longer breastfeeding duration [66].

Challenges and barriers in the NICU

Environmental Factors

The implementation of KMC is influenced by multiple interrelated factors, including healthcare system design, facility infrastructure, availability of supplies, and institutional resources. Firstly, in hospital settings, “facility conditions” typically refer to tangible infrastructural elements such as space and privacy. Studies have identified that the lack of adequate physical space and seclusion directly impedes the practice of KMC, while the availability of designated, private, and well-equipped areas significantly facilitates its application [67,68].

Secondly, the presence of clear clinical guidelines or standardized protocols within healthcare facilities plays a crucial role. The absence of such operational frameworks is among the most commonly reported barriers to KMC adoption. However, when hospitals implement formal protocols and visually promote KMC through posters or educational materials, adherence and execution of KMC practices improve substantially [69].

Thirdly, the broader healthcare system components, specifically policies and staff education, act as foundational determinants. Implementation is often hindered by restrictive institutional policies, lack of managerial support, and inconsistent or inadequate training among healthcare providers. In some settings, healthcare professionals offered insufficient guidance to mothers, and hospital staff occasionally discouraged family involvement. This sometimes led to negative perceptions of healthcare workers as unapproachable or unsympathetic [69].

Finally, key enablers for successful KMC and early KMC implementation include the formal integration of KMC principles into national health education curricula and the establishment of regulatory policies promoting its adoption. These systemic supports are essential for sustaining KMC practices, especially in low-resource or high-demand clinical environments [69].

Barriers to KMC/early KMC at healthcare institutions were noted in various studies [70]. Based on a study in Australia, it has been observed that nurses' practical problems in KMC practice include a high workload, a lack of organizational support, and unclear criteria (such as unstable babies) [71]. A study conducted in the USA discovered that low staffing and ambiguous terminology among nurses hampered the adoption of KMC, along with a lack of equipment, opposition to altering the staffing plan, and nurses [72,73]. According to one study in Brazil, KMC is limited by the preterm age of neonates, a noisy NICU, and prejudice toward job responsibility among medical professionals. The healthcare hurdles listed above suggest that healthcare professionals are not implementing early KMC at a high rate [74].

Social Barriers to Early KMC Implementation

The idea and experience that one receives help from others to carry out KMC is known as social support. Mothers and fathers did not believe that their communities or relatives supported them while they practiced KMC. Due to the prevalent belief that childcare is primarily the responsibility of mothers, many men have expressed discomfort with engaging in KMC due to societal norms. Additionally, they reported experiencing inadequate support from society [75]. KMC was not considered a suitable approach for caring for newborns by older generations, especially mothers-in-law and grandparents.

On the other hand, the presence of involved healthcare professionals, family, and community approval of early KMC and societal acceptance of a father's involvement in childcare all encouraged early KMC uptake. Fathers practicing KMC faced fewer obstacles in communities with more equitable gender roles [49]. Early KMC uptake was significantly influenced by paternal engagement, either through labor division, which refers to the distribution of caregiving responsibilities among family members, healthcare providers, and the broader community to support the effective implementation of KMC practices [59]. This concept is particularly emphasized in a narrative review [75]. Having someone to help with housework and the infant, like sisters or grandmothers, was appreciated by moms during early KMC and made them feel at ease. Furthermore, the application of KMC was made easier by the presence of skilled nurses, who decreased maternal anxiety about holding their newborn and performing KMC [76].

Access Factors

Access factors refer to the ability of both parents and healthcare providers to utilize and offer KMC services, encompassing considerations such as financial resources, time availability, and location [67]. Financial constraints play a significant role, as many individuals cannot afford extended hospital stays or private rooms required to practice early KMC after childbirth. Time is another significant barrier, particularly for healthcare staff who often struggle to allocate time for KMC training. Many perceive that implementing early KMC may increase their workload and reduce the time available for attending to other critically ill patients [49]. For parents of preterm infants, long commutes and domestic responsibilities can also hinder their ability to consistently participate in early KMC [68]. Additional challenges include transportation costs, the expense of temporary lodging, and the logistical difficulties of initiating KMC within clinical wards. Facilitating early KMC can be supported by reducing hospital-related expenses for families, minimizing overall healthcare system costs, and ensuring unrestricted visitation policies.

Awareness and Education

Lack of effective leadership, poor professionalism of workers, and insufficient training are the key barriers to early KMC implementation. A study on the implementation of KMC in Indonesian hospitals discovered that government backing, hospital administration, staff acceptance, and training were important facilitators of KMC adoption [77]. In some places, the government provided KMC-specific training programs for medical workers. Another technique for overcoming early KMC implementation issues is to educate healthcare professionals and mothers about the benefits of early KMC, as well as to implement clear rules for encouraging early KMC. Another author proposed focusing on the newborn's and mother's needs (e.g., interactional communication, increasing emotional bonding, and maternal-newborn safety) and working with healthcare professionals. This interaction provides a solid foundation for continuity of care. Perceived barriers might be reduced and early KMC practice increased using management and training initiatives, such as ongoing education between mothers and nurses on the value of early KMC. Koopman et al. [72] advised quality KMC programs in the labor and delivery units. These programs involve setting up focus groups and giving nursing staff members a platform to express their thoughts and difficulties in carrying out KMC practice [78]. In this sense, nurses are supposed to come to an agreement and assume accountability for enhancing the application of KMC/early KMC.

Family Support

Early KMC practice is hindered by a lack of support, while its application is facilitated by the support of friends, family, and other mothers. Support varied. Family members alternately held the premature infants to help the mother break this habit [79]. Research has indicated that moms benefit from emotional support in addition to assistance and support with housekeeping tasks [80]. In addition to moms, fathers, grandfathers, grandmothers, and other family members can also practice kangaroo nursing with preterm children [76]. If family members are unaware of this, preterm newborns may not be able to receive kangaroo care. As a result, several teaching strategies must be used to inform families of preterm newborns about their responsibilities within early KMC. It may be extremely difficult to train moms and support early KMC implementation in some clinical situations where mothers of preterm children have unique medical issues. According to the reviews, early KMC practice was further complicated by the medical problems of women, such as postpartum depression, postpartum recovery, post-episiotomy pain repair, and general maternal sickness [79]. Mothers may also experience mental challenges with early KMC techniques, such as posture problems (difficulty resting on the breast with newborns), complications linked to breastfeeding, and breast milk expression [81]. Father participation and family support are very important in this situation [82]. Mothers using KMC experienced fewer postpartum depression symptoms, despite it being a barrier to its adoption [83].

Global perspectives

Coverage, Estimates, and Implementation of Early KMC in NICUs

The WHO 2023 report provides evidence on health system intervention strategies for KMC implementation, which increases its coverage across various country income levels, facilities, or community settings [84]. Sixteen research articles (13 from low-to-medium-income countries and 3 from high-income settings) clearly indicate that in recent years KMC coverage has increased by at least 25% from baseline and final coverage of more than 50%, along with achieved SSC of at least eight hours/day. However, in several cases, it is observed that high-intensity interventions across multiple health systems (building blocks) should be used for maximum scale-up of KMC to achieve SSC of eight hours/day. Studies and implementation strategies included in this review aimed to increase KMC coverage and address barriers to its adoption. Relevant sources include both quantitative studies, which evaluated the effectiveness of interventions, and guidelines, which provided structured implementation frameworks. For example, studies such as Smith et al. [66] demonstrated the impact of community-based KMC programs on neonatal outcomes, while WHO guidelines [39,84] outlined practical steps for integrating KMC into routine newborn care services. These sources were included for their direct relevance to improving KMC uptake and informing policy and practice are summarized in Table 2 [84]. 

**Table 2:** Studies and implementations to increase KMC coverage and overcome barriers KMC: kangaroo mother care.

Health system building block	Important barriers identified	Key interventions that are effective in improving KMC coverage
Leadership and policy	Lack of priority and leadership support within the health system. Policy changes are not made to secure the hospital's introduction of KMC, which is only recommended in national guidelines. Male family members are not allowed inside wards to practice or support KMC.	High-level leadership engagement (national/district policymakers and program managers, and facility leadership). KMC-supportive policies by providing a bed, food, water, toilet, etc. Allow the family to support the mother in providing KMC and incorporate KMC in licensing standards for healthcare facilities.
Health financing	No separate financial allocation to cover the implementation costs of KMC for preterm or LBW infants within healthcare facilities.	KMC establishing costs are included in national and regional plans and budgets. Expanded health insurance to cover care for small and/or sick infants.
Health workforce	No dedicated KMC nursing staff; resident doctors, interns, and nurses not oriented on the practical aspects of KMC. Nurses are unable to spend time on KMC counseling due to rotations and multiple tasks. Low doctor-to-patient and nurse-to-patient ratios; lack of confidence and training of staff. Healthcare staff, including nurses, are reluctant to change old practices because of concerns about the safety of preterm or LBW infants and fear of complications.	Adequate nursing staff with strengthened competency and motivation to support KMC. Engagement of professional organizations, KMC champions, and maternal-neonatal staff collaboration.
Service delivery	Lack of counseling of mothers and relatives leads to no early initiation of KMC. Lack of interest from mothers and poor support from family, early discharge demands.	KMC wards with a conducive environment (infrastructure, support, and counseling). Community engagement to promote KMC. Early identification and facilitated referral of LBW infants.
Health information systems	Lack of regular audits of KMC practices and documentation in registers. KMC registers are not well-maintained, and data entry is not consistent.	Recording KMC (KMC-specific) indicators in routine data systems.
Supplies	Non-availability of KMC bags and binders,	KMC beds, chairs, and garments/binders.

International Guidelines and Recommendations for Early KMC in the NICU

WHO encourages nations to offer early KMC to tiny and/or ill neonates as part of an all-inclusive package of crucial, evidence-based interventions or services. WHO recommendations for the treatment of preterm or LBW newborns, postnatal care for women and infants, hospital care for children, and antenatal steroids are pertinent guidelines that may be taken into account when planning the implementation of KMC/early KMC [85]. WHO guidelines on maternal and infant health promotion interventions provide additional pertinent guidelines for programmatic implementation. Among these is the participation of dads, partners, and other family members, which is essential for early KMC since it gives the mother time to rest and take care of herself while enabling the father, partner, and other family members to assist with the baby's care. Guidelines for enhancing and standardizing neonatal care training for current health workers, auxiliary nurses, and midwives are also part of WHO's human resource strategies to improve infant care in healthcare institutions in low- and middle-income nations [86]. New research, however, indicates that community health workers can effectively instruct and assist mothers in starting and implementing KMC at home if they are given the right training. For best results, we encourage nations to include KMC in their maternal-newborn care packages and to update them frequently to reflect the most recent evidence-based recommendations [87]. Recently a pilot study of early KMC studies on newborn care in resource-limited areas of China revealed that optimizing institutional regulations, with necessary supporting resources along with education training, may help to refine the implementation and scale-up of early KMC practice in China and other parts of the world [87]. Another parallel study is the first available evidence of the potential effects of early KMC on neurological development in preterm infants, extending well beyond infancy into young adulthood. Hence, early KMC can be implemented as a powerful tool to supplement standard neonatology care as well as the overgrowth of preterm infants for better key outcomes, particularly neurodevelopment [88].

## Conclusions

Early KMC, rooted in the need for low-cost, high-impact interventions in neonatal care, has evolved into a globally recognized strategy for improving outcomes in preterm and LBW infants. This review has examined the historical development of early KMC, described its incorporation into contemporary NICUs, and analyzed the various challenges affecting its broader implementation. Despite robust evidence supporting its clinical benefits, such as improved thermoregulation, breastfeeding, bonding, and survival, barriers persist at institutional, cultural, and infrastructural levels. Continued efforts are required to address these challenges through policy support, staff training, and parent education. Moving forward, the successful scaling of early KMC will depend on a holistic approach that bridges evidence with practice, ensuring that this life-saving intervention is both accessible and sustainable across diverse healthcare settings. Further, this study adds new insights into the current literature describing barriers to and challenges of early KMC-related practices. Various models need to be developed that could provide further direction for researchers, programmers, policymakers, and health workers concerning early KMC implementation and scale-up at national and regional levels. The successful scale-up of early KMC at all levels of facility care and in the community is essential to achieving the Sustainable Development Goals for the survival, health, and well-being of preterm or LBW infants, both ill and healthy. Based on new evidence of successful scale-up of early KMC in programmatic settings from multiple countries, as well as the combined knowledge and experience of global stakeholders, this implementation plan aims to stimulate a new vision where families and parents are at the center of raising and caring for mothers and babies from birth.
